# Rhinitis as an associated factor for anxiety and depression amongst adults^☆^

**DOI:** 10.1016/j.bjorl.2016.05.008

**Published:** 2016-06-16

**Authors:** Martín Bedolla-Barajas, Jaime Morales-Romero, Norma Angélica Pulido-Guillén, Martín Robles-Figueroa, Brenda Renata Plascencia-Domínguez

**Affiliations:** a Hospital Civil de Guadalajara “Dr. Juan I. Menchaca”, Serviço de Alergia e Imunologia Clínica, Colonia La Perla, Guadalajara, Jalisco, Mexico; b Universidad Veracruzana, Instituto de Salud Pública, Colonia Industrial Ánimas, Xalapa, Veracruz, Mexico; c Universidad Tecnológica de Guadalajara, Licenciatura en Psicología, Guadalajara, Jalisco, Mexico; d Hospital Civil de Guadalajara “Dr. Juan I. Menchaca”, Especialidad en Medicina Interna, Colonia La Perla, Guadalajara, Jalisco, Mexico; e Universidad de Guadalajara, Centro Universitario de Ciencias de la Salud, Colonia Independencia Oriente, Guadalajara, Jalisco, Mexico

**Keywords:** Anxiety, Depression, Allergic rhinitis, Adults, Ansiedade, Depressão, Rinite alérgica, Adultos

## Abstract

**Introduction:**

Anxiety and depression are frequent disorders of chronic diseases, yet there is no conclusive information to their association with rhinitis.

**Objective:**

The objective is to determine the frequency of anxiety and depression and its possible association to allergic rhinitis (AR) and non-allergic rhinitis (NAR).

**Methods:**

This is a cross-sectional study in which procured subjects with AR (*n* = 111), NAR (*n* = 34) and a control group (*n* = 96) from the university hospital. The presence of anxiety and depression was considered when it reached a score > 13 based on The Beck Anxiety Inventory Test and The Beck Depression Inventory II Test, respectively. The association between AR and NAR with anxiety and depression was adjusted with the Mantel–Haenszel Method and logistic regression.

**Results:**

The frequency for anxiety in AR, NAR and the control group was 45.9%, 52.9%, 10.4%, respectively (*p* < 0.001); depression frequency was 38.7%, 47.1%, 16.6% (*p* = 0.0003), respectively. Both AR and NAR were associated to anxiety and depression in women, but not to men. After adjusting the sex: AR was associated to anxiety (OR = 5.7, *p* < 0.001) and depression (OR = 2.5, *p* = 0.015), while NAR was also associated to anxiety (OR = 7.8, *p* < 0.001) and depression (OR = 3.3, *p* < 0.014). In multivariate analysis it was identified that AR, NAR and the individual's sex (women) were factors associated to anxiety and depression. Results showed that age was only associated to anxiety.

**Conclusion:**

AR and NAR are diseases associated to anxiety and depression, at least in women.

## Resumo

**Introdução:**

Ansiedade e depressão são distúrbios frequentes em doenças crônicas, embora não haja nenhuma informação conclusiva à sua associação com a rinite alérgica.

**Objetivo:**

O objetivo do estudo é determinar a frequência de ansiedade e depressão e sua possível associação a rinite alérgica (RA) e rinite não alérgica (NRA).

**Método:**

Estudo transversal, no qual indivíduos com RA (n = 111), NRA (n = 34) e um grupo controle (n = 96) foram recrutados a partir do hospital universitário. A presença de ansiedade e depressão foi considerada quando atingiu uma pontuação > 13 no Teste do Inventário de Ansiedade de Beck e no Teste II do Inventário de Depressão de Beck, respectivamente. A associação entre a RA e RNA com ansiedade e depressão foi ajustada com o Método de Mantel-Haenszel e regressão logística.

**Resultados:**

A frequência de ansiedade na RA, RNA e grupo controle foi de 45,9%, 52,9%, 10,4%, respectivamente (*p* < 0,001); a frequência de depressão foi de 38,7%, 47,1%, 16,6% (*p* = 0,0003), respectivamente. Tanto a RA como a RNA foram associadas a ansiedade e depressão em mulheres, mas não para os homens. Depois de ajustar o sexo: RA foi associada à ansiedade (OR = 5,7, *p* < 0,001) e depressão (OR = 2,5, *p* = 0,015), enquanto a RNA foi associada a ansiedade (OR = 7,8, *p* < 0,001) e depressão (OR = 3,3, *p* < 0,014). Na análise multivariada identificou-se que a RA, RNA e o sexo do indivíduo (mulheres) foram fatores associados a ansiedade e depressão. Os resultados mostraram que a idade só foi associada à ansiedade.

**Conclusão:**

RA e RNA são doenças associadas a ansiedade e depressão, pelo menos em mulheres.

## Introduction

By definition, rhinitis is an inflammatory alteration of the nasal mucus; which is also characterized by the presence of two or more of the following symptoms: nasal congestion, rhinorrhea, itchiness, sneezing, and daily alterations in a patients sense of smell for over an hour.^1^ By classifying the presence or absence of allergic sensibilization, it is possible to differentiate between allergic rhinitis (AR) and non-allergic rhinitis (NAR).^2^

Amongst the long evolution rhinitis, highlights the AR, disorder causing significant morbidity and disability, which affects the quality of life in social, school and workplace; causes a high economic impact as well as an increase in the consumption of alcoholic beverages and sedatives.^2^, ^3^, ^4^, ^5^, ^6^ Additionally, this disrupts sleep patterns, along with cognitive and productive capacities.^3^, ^5^ Up to 43% of AR patients wake up feeling tired, this is reflected in matters such as their mood and sexual appetite, furthermore, it has been reported as a risk factor for depression and anxiety.^5^, ^7^, ^8^

The mood disorder characterized by sadness, feelings of uselessness, self-depreciation, and social isolation, are known as depression; anxiety is the feeling of restlessness or apprehension that acts as a defense mechanism and aids in managing potential danger, this appears to have an adaptive function as it produces bodily reactions that can prepare an individual for the “fight or flight” response.^9^

The disorder characterized by anxiety and mood changes are common in patients with chronic diseases,^10^, ^11^, ^12^, ^13^ however, in the case of rhinitis, these side effects have not yet been researched thoroughly. For a more in-depth understanding of these associations, more clinical evidence is needed; thus far, most studies concerning anxiety and depression as possible consequences of rhinitis have been epidemiological.^14^, ^15^, ^16^, ^17^

The objectives in this study consisted of determining the levels of anxiety and depression from a group of patients with AR and another group with NAR; thereafter, we compared our results with a group of control subjects. This was carried out in order to obtain a greater understanding of the association that rhinitis has to anxiety and depression.

## Methods

### Ethics

The Hospital's Ethical Research Committee approved this study (number: 1226/12). Each subject signed a written consent form in order to participate, additionally; every individual was informed that if he or she declined to participate, it would not affect the quality of his or her medical treatment. Immediately thereafter we obtained results from the tests that identified anxiety and depression, all subjects were informed of their results, and when necessary, they were offered specialized psychological attention.

### Design

A cross-sectional study was carried out amongst patients with AR symptoms that sought external allergy consultations in a university hospital; these were later compared to a group of patients with NAR and a group of healthy patients (control group).

The recruitment process was consecutive and effected from July 2012 to March 2013. AR patients were selected with the following criteria: aged ≥18 and ≤50, they had to be first time allergy consulting patients with a rhinitis diagnosis, regardless of its severity. The second and third groups, respectively, constituted by subjects with NAR (rhinitis symptoms and negative skin prick tests to aeroallergens) and healthy subjects (consisting of blood donors that were consecutively denominated as the control group) whose ages and geographic provenience matched subjects from the AR group. Subjects that lived outside the city or patients with diabetes mellitus, arterial hypertension, rheumatoid arthritis, asthma, urticaria, renal failure or neoplasias were not included in our study.

### Definitions, instruments and scales

All AR diagnoses were clinically established and carried out by a single allergy specialist. Each participant had at least one positive skin prick test to one of the aeroallergens tested, these included: grass pollens, weeds, trees, household dust mites, cat and dog epitheliums, and atmospheric fungi. AR was classified according to the frequency of the symptoms, as intermittent or persistent; and based on the severity, mild or moderate to severe, as recommended by ARIA (Allergic Rhinitis and its Impact on Asthma).^2^

Anxiety and depression symptoms were evaluated using The Beck Anxiety Inventory and The Beck Depression Inventory-II, both in the Spanish language version and validated in the Mexican population studies.^18^ These tests consist of 21 items whose sum of points obtained in each of the questions (63 maximum points possible) allow the categorization of anxiety and depression as: (a) mild (14–19 points), (b) moderate (20–28 points) and (c) severe (29–63 points).

Body mass index was determined by weighing and measuring each subject the day of the medical consultation, calculating: weight (kg)/height (m^2^).

Prior to recruiting our patients, a pilot test was carried out on five individuals with AR, they were not included in the final analysis. The pilot test allowed us to correct the selection criteria for subjects and to amend the written questionnaires.

### Statistical analyses

The clinical and demographic characteristics of each subject were expressed through means, standard deviations and proportions. The frequency of anxiety and depression, as well as their levels of severity, were expressed as percentages within each comparative group (AR, NAR, and control). Generally, independent group proportions were compared using Chi Squared tests or the Fisher's Exact Test. In order to find an association between AR or NAR and their link with anxiety or depression, the odds ratio was calculated with confidence intervals at 95%, adjusted by sex through stratified analysis using the Mantel and Haenszel method. Lastly, we used a multivariate logistic regression model to evaluate the association that allergic and non-allergic rhinitis (independent variables) have with anxiety and depression (dependent variables), while also introducing co-variants (sex and age) into the model. Any value of *p* ≤ 0.05 was considered to be statistically significant. For data analysis, we used IBM SPSS Statistics (Armonk, NY, USA), version 23.0.

## Results

This study included 241 subjects that were placed into three groups: allergic rhinitis (*n* = 111), non-allergic rhinitis (*n* = 34) and a control group (*n* = 96). The mean age for each study group was approximately 30; women were the majority of the patients with any type of rhinitis while men were predominant in the control group (Table 1). Within the control group, almost 15% of the individuals smoked tobacco, and a little over 50% consumed some sort of alcoholic beverage. Over half of the participants took part in some type of physical activity at least once a week and had an average of 7 h of daily sleep. Patients with AR had persistent clinical symptoms with moderate to severe levels, amongst these, the most common aeroallergen where pollens, principally, those originating from trees.

**Table 1:** Clinical characteristics of test subjects.

	Allergic rhinitis (*n* = 111)	Non-allergic rhinitis (*n* = 34)	Control group (*n* = 96)
*Mean age in years* *±* *SD*	32.9 ± 10.4	36.5 ± 10.4	32.1 ± 9.7
*Female, n (%)*	82 (73.9)	28 (82.4)	37 (38.5)

*Marital status, n (%)*			
Single	49 (44.1)	13 (38.2)	40 (41.7)
Married	47 (42.3)	16 (47.1)	41 (42.7)
Live-in partner	8 (7.2)	3 (8.8)	7 (7.3)
Divorced	4 (3.6)	0 (0)	5 (5.2)
Widowed	3 (2.7)	2 (5.9)	3 (3.1)

*Current tobacco intake, n (%)*	9 (8.1)	2 (5.9)	14 (14.6)
*Current alcohol intake, n (%)*	27 (24.3)	4 (11.8)	49 (51.0)

*Symptom frequency, n (%)*			
Intermittent,	28 (25.2)	–	–
Persistent	83 (74.8)	–	–

*Symptom intensity, n (%)*			
Mild	22 (19.8)	–	–
Moderate-severe	89 (80.2)	–	–

*Time of evolution, years* *±* *SD*	8.2 ± 7.1	7.5 ± 7.9	–
*BMI, kg/m*^*2*^ *±* *SD*	26.1 ± 5.7	27.4 ± 6.2	26.9 ± 3.7

*Sensibilization, n (%)*			
Trees	74 (66.7)	–	–
Grasses	57 (51.4)	–	–
Dust mites	53 (47.7)	–	–
Animal hair	53 (47.7)	–	–
Weeds	51 (45.9)	–	–
Cockroaches	41 (36.9)	–	–
Fungi	10 (9.0)	–	–

*n*, subjects with the characteristics of interest; SD, standard deviation; BMI, body mass index.

The global anxiety frequency amongst patients with AR (51/111; 45.9%) and NAR (18/34; 52.9%) was significantly higher in comparison to subjects from the control group (*p* < 0.001). Similarly, depression frequency amongst those with AR (43/111; 38.7%) and in NAR (16/34; 47.1%) was significantly higher to that of the control group (*p* = 0.0003). In the AR group, moderate levels of anxiety and depression were predominant; thus, within the NAR group, severe and moderate levels were predominant, respectively (Table 2).

**Table 2:** Frequency of anxiety and depression symptoms based on the type of rhinitis.

	Allergic rhinitis (*n* = 111)	Non-allergic rhinitis (*n* = 34)	Controls (*n* = 96)	*p*
**Anxiety,*****n*****(%)**				
*Total*	51 (45.9)	18 (52.9)	10 (10.4)	<0.001

*Severity scale*				
Severe	15 (13.5)	11 (32.4)	1 (1.0)	
Moderate	25 (22.5)	3 (8.8)	2 (2.1)	
Mild	11 (9.9)	4 (11.8)	7 (7.3)	

**No anxiety**	60 (54.1)	16 (47.0)	86 (89.6)	

**Depression,*****n*****(%)**				
*Total*	43 (38.7)	16 (47.1)	16 (16.6)	0.0003

*Severity scale*				
Severe	7 (6.3)	6 (17.6)	3 (3.1)	
Moderate	19 (17.1)	7 (20.6)	2 (2.1)	
Mild	17 (15.3)	3 (8.8)	11 (11.5)	

**Not depressed**	68 (61.3)	18 (53.0)	80 (83.3)	

*n*, subjects with characteristics of interest.

Total number of subjects with symptoms of anxiety or depression was compared to groups with allergic rhinitis and non-allergic rhinitis and the controls using the Chi Squared test.

The frequency of anxiety or depression did not differ according to the intensity and frequency of AR symptoms, Table 3.

**Table 3:** Frequency of anxiety and depression based on the classification of allergic rhinitis symptoms.

Classification	Anxiety			Depression		
	*n*	%	*p*	*n*	%	*p*
**Allergic rhinitis**						
*Symptom intensity*						
Mild, *n* = 22	7	31.8	0.138	7	31.8	0.457
Moderate-severe, *n* = 89	44	49.4		36	40.4	

*Symptom frequency*						
Intermittent, *n* = 28	13	46.4	0.953	9	32.1	0.407
Persistent, *n* = 83	38	45.8		34	41.0	

*n*, subjects with characteristics of interest.

*p*-value was obtained through the Chi Squared test.

Overall, when the AR and NAR groups were compared to the control group, there was a significantly higher frequency of anxiety: AR (OR = 7.3, *p* < 0.0001) and in NAR (OR = 9.7, *p* < 0.001). However, once stratified by sex, this association was only consistent amongst women, but not men, although amongst the latter, the association reached a significant limit. When adjusted by sex, AR and NAR maintained significant association to anxiety (Table 4). Similarly, both types of rhinitis were also associated with depression, although the level of association was much lower than that seen for anxiety (OR = 2.9, *p* < 0.001 and OR = 4.1, *p* < 0.001 for AR and NAR, respectively). Similarly, this association is not ratified in men, only women. Finally, when we weighed these groups, the association remained statistically significant after adjusting it by sex (Table 4).

**Table 4:** Risk of anxiety and depression in subjects with rhinitis adjusted by sex.

	Anxiety			Depression		
	OR	95% CI	*p*	OR	95% CI	*p*
*Allergic rhinitis*^a^						
General	7.3	3.44–15.53	<0.001	2.9	1.54–5.62	0.0009
Men	3.4	0.98–12.0	0.06	2.1	0.72–6.23	0.17
Women	7.4	2.62–20.92	<0.001	2.7	1.10–6.62	0.03
Adjusted by sex	5.7	5.58–12.44	<0.001	2.5	1.24–4.90	0.015

*Non-allergic rhinitis*^a^						
General	9.7	3.78–24.75	<0.001	4.1	1.76–9.70	0.0007
Men	5.4	0.78–37.16	0.12	1.11	0.12–10.66	0.99
Women	8.5	2.56–28.43	0.0002	4.2	1.42–12.30	0.008
Adjusted by sex	7.8	2.79–21.52	<0.001	3.3	1.28–8.25	0.014

OR, odds ratio; 95% CI, confidence intervals at 95%; OR adjusted by Mantel and Haenszel.

*p*-value obtained by using Chi Squared or Fisher's Exact Test.

a Compared vs. control group.

Table 5 shows the risk of anxiety and depression in patients with RA and RNA adjusted by multivariate analysis. When compared to the control group, both AR and NAR were identified as risk factors for anxiety and depression. Furthermore, women presented a higher risk for anxiety and depression, because age seemed to have a week significant association to anxiety, it was taken out from the model.

**Table 5:** Multivariate model of association for allergic and non-allergic rhinitis with anxiety and depression.

	Anxiety			Depression		
	OR	95% CI	*p*	OR	95% CI	*p*
Allergic rhinitis^a^	5.9	2.7–13.0	<0.001	2.5	1.3–5.0	0.009
Non-allergic rhinitis^a^	6.2	2.3–16.7	<0.001	3.3	1.4–8.2	0.009
Sex (female)	2.5	1.2–5.2	0.011	2.1	1.1–4.1	0.027
Age (years)	1.05	1.02–1.08	0.004	–	–	0.073

OR obtained through logistic regression with the Forward Conditional Method.

ORs were not calculated for variables excluded from the model.

All covariates were categorical, except age (in years), which was introduced on a continuous scale.

a Compared vs. control group.

## Discussion

Our findings highlight several noteworthy aspects: first, patients with AR and NAR had a higher prevalence for anxiety and depression than the control group. Second, our data suggests that the association that rhinitis has with anxiety and depression is specific in women. Third, rhinitis itself, and not the atopic state, is what causes alterations of psychological relevance.

Both anxiety and depression are mental health problems that appear in a higher frequency amongst individuals with diseases of prolonged evolution: chronic urticaria,^10^ rheumatoid arthritis,^11^ chronic respiratory diseases,^12^ cardiovascular diseases,^13^ however, only on a few occasions, have these been studied in chronic diseases of the nose. In our study, over a third of the tested patients with some type of rhinitis denoted the presence of anxiety or depression. These findings are consistent with previous studies, which show that subjects with AR or vasomotor rhinitis present a very high frequency of anxiety than the control group.^14^, ^19^ Our study clearly illustrates a predominant occurrence of anxiety and depression in women rather than men, with both AR and NAR. Consistently, a study based on a population of adults showed that the probability of presenting symptoms for depression was greater in women that had a history of allergies^14^; however, it seems that the atopic state is not the only link to anxiety or depression, as this association also occurs in women affected by gastrointestinal illnesses,^20^, ^21^ skin diseases^22^ or cardiovascular diseases,^13^ amongst others. The most important explanations could be found in family-life and difficult experiences during childhood, as well as a previous history of anxiety or depression, social circumstances, cultural norms and vital difficulties.^23^ It is necessary to carry out further studies to better understand the role that gender plays in the genesis of psychological disorders.

There are previous studies that suggest there is a link between anxiety and depression to allergies. Buske-Kirschbaum et al.^24^ observed that patients with atopic dermatitis or allergic rhinitis shared similarities in personality traits. More recently, in a population study Goodwin et al.^15^ highlighted that subjects with allergic diseases had a higher frequency of anxiety disorders and mood swings when compared to patients without allergies. This suggests that atopy is associated to psychological alterations and not to the chronic evolution of the disease. To better clarify this situation, we compared a group of patients with AR and another with NAR, whose symptoms are indistinguishable. Surprisingly, we observed that in both rhinitis groups anxiety and depression were closely associated. Patients with a diminishing or a lost sense of smell, which tend to be followed by processes of chronic nasal inflammation, have been associated to high frequencies of anxiety or depression.^25^, ^26^ Another factor that often accompanies nose diseases is inflammation; the products that are released during this process could explain the origins of depression and anxiety.^27^

Notwithstanding, we found no association between the severity of AR to anxiety or depression. The information is inconsistent since one population study only showed association when AR was moderate to severe and persistent,^28^ another study carried out on adolescents with allergic rhinitis did not find any evidence of association.^29^ It has been proposed that the individual's attitude toward the illness is what determines the levels of allergic severity and symptoms for anxiety and depression.^30^

### Limitations

When interpreting the following results, it is necessary to consider these limitations. First, the lack of confirmation of the diagnosis of anxiety and depression by direct interview. Instead, we use an instrument that has been validated in our population and strongly correlates with concepts proposed by DSM-IV. Also worth noting that both Beck inventories measure the presence of anxiety and depression in the weeks before and not during the past 12 months or in some stage of life as they have done other studies. Second, the difficulty to generalize our results to other age groups, since we decided to evaluate only adults 18–50 years old. Third, the difficulty of assessing the influence of drugs used in controlling the symptoms of rhinitis, where it has been notified that some of them are related to anxiety disorders and depression. Four, we did not evaluate the possible association that anxiety or depression have in different levels of severity for NAR, as we did not have a scale to reference. Lastly, the present study has its own cross-sectional study limitations, since it is impossible to determine if the rhinitis precedes the anxiety or the depression.

## Conclusion

We conclude that patients with AR and NAR, particularly women, have a higher frequency of anxiety and depression when compared healthy subjects. It is likely that the cause of this association is the chronic nature of the disease and not the atopy itself.

## Conflicts of interest

The authors declare no conflicts of interest.

## References

[1] Sacre Hazouri J.A. (2010). Non-allergic chronic rhinitis. Rev Alerg Mex.

[2] Cagnani C.E., Solé D., Díaz S.N., Zernotti M.E., Sisul J.C., Borges M.S. (2009). Allergic rhinitis update and its impact on asthma (ARIA 2008). Latin American perspective. Rev Alergia Mex.

[3] Camelo-Nuñes I.C., Solé D. (2010). Allergic rhinitis: indicators of quality of life. J Bras Pneumol.

[4] Dávila I., Mullol J., Ferrer M., Bartra J., del Cuvillo A., Montoro J. (2009). Genetic aspects of allergic rhinitis. J Investig Allergol Clin Immunol.

[5] Léger D., Annesi-Maesano I., Carat F., Rugina M., Chanal I., Pribil C. (2006). Allergic rhinitis and its consequences on quality of sleep: an unexplored area. Arch Intern Med.

[6] Manalai P., Hamilton R.G., Langenberg P., Kosisky S.E., Lapidus M., Sleemi A. (2012). Pollen-specific immunoglobulin E positivity is associated with worsening of depression scores in bipolar disorder patients during high pollen season. Bipolar Disord.

[7] Gauci M., King M.G., Saxarra H., Tulloch B.J., Husband A.J. (1993). A Minnesota Multiphasic Personality Inventory profile of women with allergic rhinitis. Psychosom Med.

[8] Postolache T.T., Komarow H., Tonelli L.H. (2008). Allergy: a risk factor for suicide?. Curr Treat Options Neurol.

[9] Sue D., Sue D.W., Sue S. (2012). Psicopatología Comprendiendo la conducta anormal.

[10] Staubach P., Dechene M., Metz M., Magerl M., Siebenhaar F., Weller K. (2011). High prevalence of mental disorders and emotional distress in patients with chronic spontaneous urticaria. Acta Derm Venereol.

[11] Amira O. (2011). Prevalence of symptoms of depression among patients with chronic kidney disease. Niger J Clin Pract.

[12] Ryu Y.J., Chun E.M., Lee J.H., Chang J.H. (2010). Prevalence of depression and anxiety in outpatients with chronic airway lung disease. Korean J Intern Med.

[13] Kilzieh N., Rastam S., Maziak W., Ward K.D. (2008). Comorbidity of depression with chronic diseases: a population-based study in Aleppo, Syria. Int J Psychiatry Med.

[14] Goodwin R.D., Castro M., Kovacs M. (2006). Major depression and allergy: does neuroticism explain the relationship?. Psychosom Med.

[15] Goodwin R.D., Galea S., Perzanowski M., Jacobi F. (2012). Impact of allergy treatment on the association between allergies and mood and anxiety in a population sample. Clin Exp Allergy.

[16] Sanna L., Stuart A.L., Pasco J.A., Jacka F.N., Berk M., Maes M. (2014). Atopic disorders and depression: findings from a large, population-based study. J Affect Disord.

[17] An S.Y., Choi H.G., Kim S.W., Park B., Lee J.S., Jang J.H. (2015). Analysis of various risk factors predisposing subjects to allergic rhinitis. Asian Pac J Allergy Immunol.

[18] Beltrán M., Freyre M., Hernández L. (2012). The Beck Depression Inventory: its validity in adolescent population. Terapia Psicol.

[19] Addolorato G., Ancona C., Capristo E., Graziosetto R., Di Rienzo L., Maurizi M. (1999). State and trait anxiety in women affected by allergic and vasomotor rhinitis. J Psychosom Res.

[20] Addolorato G., Mirijello A., D’Angelo C., Leggio L., Ferrulli A., Abenavoli L. (2008). State and trait anxiety and depression in patients affected by gastrointestinal diseases: psychometric evaluation of 1641 patients referred to an internal medicine outpatient setting. Int J Clin Pract.

[21] Alosaimi F.D., Al-Sultan O.A., Alghamdi Q.A., Almohaimeed I.K., Alqannas S.I. (2014). Gender-specific differences in depression and anxiety symptoms and help-seeking behavior among gastroenterology patients in Riyadh, Saudi Arabia. Neurosciences (Riyadh).

[22] Annesi-Maesano I., Beyer A., Marmouz F., Mathelier-Fusade P., Vervloet D., Bauchau V. (2006). Do patients with skin allergies have higher levels of anxiety than patients with allergic respiratory diseases? Results of a large-scale cross-sectional study in a French population. Br J Dermatol.

[23] Piccinelli M., Wilkinson G. (2000). Gender differences in depression. Critical review. Br J Psychiatry.

[24] Buske-Kirschbaum A., Ebrecht M., Kern S., Gierens A., Hellhammer D.H. (2008). Personality characteristics in chronic and non-chronic allergic conditions. Brain Behav Immun.

[25] Di Rienzo Businco L., Di Rienzo Businco A., Lauriello M., Coen Tirelli G. (2004). State and trait anxiety in patients affected by nasal polyposis before and after medical treatment. Acta Otorhinolaryngol Ital.

[26] Katotomichelakis M., Simopoulos E., Tzikos A., Balatsouras D., Tripsianis G., Danielides G. (2014). Demographic correlates of anxiety and depression symptoms in chronic sinonasal diseases. Int J Psychiatry Med.

[27] Müller N., Ackenheil M. (1998). Psychoneuroimmunology and the cytokine action in the CNS: implications for psychiatric disorders. Prog Neuropsychopharmacol Biol Psychiatry.

[28] Kim D.H., Han K., Kim S.H. (2016). Relationship between allergic rhinitis and mental health in the general Korean adult population. Allergy Asthma Immunol Res.

[29] Adamia N., Jorjoliani L., Manjavidze N., Ubiria I., Saginadze L. (2015). Psycho-emotional characteristics of the adolescents with allergic rhinitis. Georgian Med News.

[30] Molzon E.S., Suorsa K.I., Hullmann S.E., Ryan J.L., Mullins L.L. (2012). The relationship of allergy severity to depressive and anxious symptomatology: the role of attitude to ward illness. ISRN Allergy.

